# Inhibited and Retarded Behavior by Ca^2+^ and Ca^2+^/OD Loading Rate on Ureolytic Bacteria in MICP Process

**DOI:** 10.3390/ma16093357

**Published:** 2023-04-25

**Authors:** Masaharu Fukue, Zbigniew Lechowicz, Yuichi Fujimori, Kentaro Emori, Catherine N. Mulligan

**Affiliations:** 1 Japanese Geotechnical Association for Housing Disaster Prevention, 1622, Oshikiri, Shimizu-ku, Shizuoka 424-0008, Shizuoka, Japan; fukue@scc.u-tokai.ac.jp; 2 Department of Geotechnical Engineering, Institute of Civil Engineering, Warsaw University of Life Sciences, Nowoursynowska 159, 02-776 Warsaw, Poland; zbigniew_lechowicz@sggw.edu.pl; 3 Chubu Sokuchi Research Institute Co., 801-1 Konami, Suwa City 392-0131, Nagano, Japan; fujimori-yuichi@chubusokuchi-lab.co.jp; 4 Sanko Kaihatsu Co., Ltd., 1320 Gokanjima, Fuji City 416-0946, Shizuoka, Japan; k.emori@sankoukaihatsu.co.jp; 5 Department of Building, Civil and Environment Engineering, Concordia University, 1455 de Maisonneuve Blvd. W., Montreal, QC H3G 1M8, Canada

**Keywords:** MICP, inhibition, retarded reaction, viability of cells, OD-conversion, Ca^2+^ loading

## Abstract

The estimation of optical density (OD) with viable cells is challenging for engineering purposes. In this study, the OD conversion based on previous study was used. The inhibited and retarded behavior of the microbially induced carbonate precipitation (MICP) process was examined. The experimental results showed that high Ca^2+^ drastically influences the inhibited and retarded behavior on MICP processes. The analysis showed that the inhibition and retardation effects occurred when the Ca^2+^/OD loading rate exceeded 8.46 M. The critical value was equal to the proportional constant for obtaining carbonate precipitation rate (CPR) from OD. Due to this, the blending design of materials became possible, with no risk of inhibition. In conclusion, the inhibition and retardation of the MICP process are governed by the Ca^2+^ load and the linear standard line (LSL), which may be attributed to the capacity or tolerance of viable cells, i.e., CPR/OD = 8.46 M or Ca^2+^/OD = 8.46 M.

## 1. Introduction

Recently, studies of microbially induced carbonate precipitation (MICP) technology were made for various objectives [1,2,3,4,5,6,7]. In soil improvement, the compressive strength of biocemented soils has been measured to investigate the effects of carbonate content and the applicability for actual practice [8,9,10,11,12,13]. The problem arising in those studies was how to control the carbonate precipitation rate (CPR). It was pointed out that in many cases, the intended MICP characteristics have not been satisfied for many reasons. Then, Lai et al. [14] focused on the retardation effects due to high Ca^2+^, while Cui et al. [15] suggested that high urease activity is important for decreasing inhibition or retardation. On the other hand, it was found that the overload of Ca^2+^/OD caused the inhibition and retardation of MICP [16].

In the MICP technology, the numbers of alive cells to be used can be one of the most crucial factors which govern the carbonate precipitation process [16]. The cell viability presents the quantity of urease, which influences the total urease activity. The urease activity governs the decomposition rate of urea in water. As a result, the carbonate precipitation induced can be influenced by the cell viability [15,16,17,18]. A sufficient viability of cell results in the full precipitation of carbonate under the concentration of Ca^2+^ was provided, and relatively low viability may result in the retarded reaction. Thus, Ca^2+^/OD ratio should be a good parameter to describe the properties and behavior concerning Ca^2+^ and microbes for the MICP process. The overloading effects observed by Fukue et al. [16] can be associated with a critical Ca^2+^/OD ratio as the condition for the inhibition and retardation of MICP.

The influence of cell viability on the carbonate precipitation has not been well understood because there has not been any practical methods for measuring cell viability for engineering purposes. In microbiology, cell numbers are usually represented by the optical density, OD_600nm_. However, the cell number does not represent cell viability for aged cells because cell viability decreases with age and other factors such as temperature including heat and cold shock and pH change.

In microbiology, the colony forming unit, CFU, has been used as the method to count the numbers of alive cells [17,19]. However, the CFU method takes time and requires technical knowledge in microbiology. Accordingly, using the CFU method has been a major obstacle to the development of MICP technology for engineering purposes [17,19].

At present, only OD_600nm_ gives information on bacteria cells for the study of engineering purposes. However, the OD_600_ is not constant, but varies day to day, as bacteria strains are damaged with time. Furthermore, there is a disadvantage in using OD_600_ in that the dead cells are also counted.

Thus, the OD of aged bacteria does not provide information on the quality of the viable cells. Therefore, the quantity and quality of in situ cultivated microbes are difficult to obtain. In other words, the OD of aged microbes does not represent viable OD but the turbidity of suspended matter. However, there is a way to change this from a disadvantage to an advantage. By using the OD value, this can be achieved by defining the following relationships [16]:

OD = Rcv · OD*(1)

where OD is defined as the viable OD, and Rcv is the viability and OD* is the tentative OD of solution with aged cells. Note that the Rcv decreases with time and with changes in pH and temperature [16]. For engineering purposes, simple and effective test techniques to evaluate the quality of bacteria solution are desired.

Similar to Equation (1), the following relationship is defined in this study:

OD = Df · OD_0_ (2)

where OD_0_ is defined as an OD equal to 1.0 in the standard solution [16]. The viability of the standard solution with an OD = 1.0 is also assumed as unity, i.e., 100%, and Df is the dilution factor. Therefore, OD varies from 1.0 to 0 as a function of Df. For example, if Df = 0.7, the corresponding OD is 0.7. Thus, any OD-solution can be prepared by the dilution process.

In this study, the existence of OD-CPR as a standard relationship, i.e., viable OD carbonate precipitation rate is the most significant hypothesis. This means that the MICP process is a function of the number of viable cells in solution. As far as CPR is a function of OD, OD becomes a representative of CPR. In fact, this OD–CPR relationship was defined as the standard OD–CPR relationship, 24 h SC, under a given condition [16]. Experimentally, the relationship without inhibition was obtained as a linear function,

CPR = 8.46 · OD(3)

and with retardation effects,

CPR = −17.633 · OD^2^ (4)

Combining Equations (3) and (4)

CPR = 8.46 · OD − 17.633 · OD^2^ (5)

where Equation (3) is graphically expressed by the linear standard line, LSL.

Substituting Equation (1) into (5),

CPR = 8.46 · (Rcv · OD*) − 17.633 · (Rcv · OD*)^2^ (6)

where (Rcv OD*) is used to identify viable OD of aged unknown bacteria. Generally, the OD of the biocement solution is unknown, but Equation (6) is utilized in tests, where OD* is basically arbitrary. Equating Equation (5) with Equation (6), Rcv can be experimentally determined. Once Rcv is determined, OD is also determined as Rcv OD*.

In MICP studies, the quality of microbes has been discussed in terms of urease activity [20,21,22,23,24,25,26,27]. The urease activity or OD are the trigger for the MICP process, which is given by the following reaction:

(NH_2_)_2_CO + [e] + H_2_O → 2NH_3_ + CO_2_ (7)

Reaction (7) is well known as hydrolysis with the catalysis of urease [e], in which the first products are NH_3_ and CO_2_ [22]. The 2NH_3_ and CO_2_ can easily be transported to the outside of cells, and react with H_2_O, as follows:

2NH_3_ + CO_2_ + H_2_O → 2(NH_4_)^+^ + CO_3_^2−^ (8)

where the urease activity of biomass increases proportionally with the numbers of cells. This implies that the concentration of microbes plays a key role in the generation rate of carbonate ions.

Accordingly, the concentration of Ca^2+^ becomes a dominant factor for the CaCO_3_ precipitation rate. However, this type of reaction is not characterized by the controlled type of biomineralization (Reaction (9)), but the induced type [27].

Ca^2+^ + CO_3_^2−^ → CaCO_3_ (9)

The surface of the cell is negatively charged and surrounded by cations, i.e., Ca^2+^. The interface between the cells and electrolyte solution forms the diffuse double layer [28,29]. It leads to a simple question regarding whether a high concentration of cation can inhibit or retard the movement of chemicals. Therefore, this study was selected as a new avenue following a previous article [16]. For example, if this is the case then the intake of urea into the cell or the discharge of CO_2_ and NH_3_ can be inhibited or retarded by the diffuse double layer.

**Figure 1:**
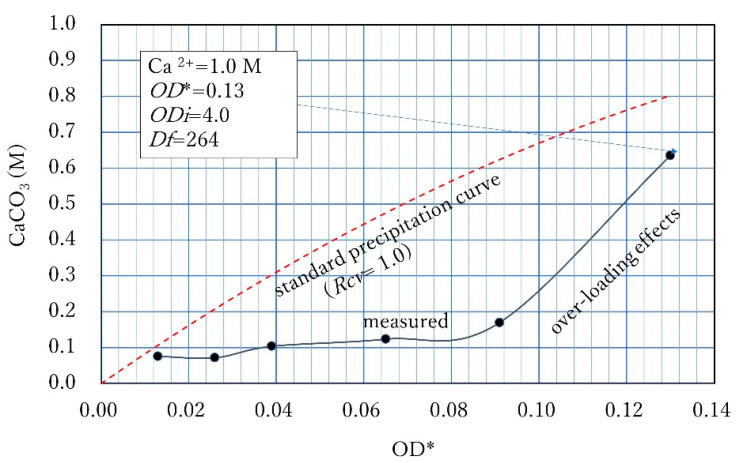
Unified OD and CPR relationship for relatively high Ca^2+^ under the condition of OD* = OD [16].

Figure 1 shows a test result which was conducted to determine an Rcv value of aged frozen bacteria. It is one of the cases where the experimental results obtained were not expected. The intended result was the standard precipitation curve shown in Figure 1. In fact, Figure 1 was presented in the previous article to demonstrate 1.0 M Ca^2+^ overload effects. The experimental elapsed time was 24 h [16]. It is also a fact that the standard OD-CPR curve, 24 h SC, is obtained by the decreased Ca^2+^ load.

One of the objectives in this study is to understand the condition of the inhibition and retardation on the MICP process. It is noted that the analyses due to the OD conversion may be a new aspect for the MICP study.

It is important to note that in Figure 1, the standard precipitation curve and the overload data are regarded as those after the OD conversion. This means that the data showing retardation and inhibition do not accord with the standard precipitation curve. In other words, the data without inhibition and retardation data after OD-conversion accord with the 24 h SC. Therefore, it is possible to judge whether or not the data are from the usual MICP process or from the 24 h SC data. This is discussed later.

## 2. Materials and Methods

### 2.1. NO-A10 Strains

In the previous studies, to prepare the assumed OD* of the biocement solution, BCS, the following relationship was used for various states of the bacteria stored in a freezer:

ODi · V_bac_ · Cf = OD* · V_BC_ (10)

where ODi is the initial OD of fresh cells in the cultivated solution, V_bac_ is the thawed volume of concentrated bacteria required to prepare the OD* solution, Cf is the concentration factor due to centrifugation and V_BC_ is the volume of the BC solution.

Thus, the optimization of the blending of materials is important to avoid the failure of engineering design, although this has not been established yet. In addition, it is also important from the economic and environmental point of view. In this study, the inhibition mechanism by Ca^2+^ on bacteria is experimentally studied to establish the design for biocement technology.

The bacteria used in this study were isolated from the alluvial soils in Japan in 2008, which were named as NO-A10, and the microbiological aspects have been described in the literature where the nucleotide sequence of NO-A10 showed 93% similarity to *S. pasteurii*, which is often used by other researchers [30]. A batch system for 300 L cultivation used a cell incubator with a volume of 1 m^3^. A 100 L of culture liquid was continuously centrifuged at 4 °C. The centrifugal concentration factors are usually 250 to 300. The centrifuged bacteria are quickly frozen and stored in a freezer at −80 °C until utilized.

### 2.2. Reactive Agents

The reactive agents used were urea, calcium chloride, distilled water and ammonia buffer. These agents are those being used for MICP techniques due to the ureolytic bacteria [16]. The bacteria were added into the reaction solution just before injection. In this study, the blending of the agents was varied to investigate the effects of Ca^2+^ on bacteria and CPR. The maximum concentration of Ca^2+^ used was 1.0 M and the 1.1 M urea was used. A 20 mM ammonia buffer solution was used for the biocement solution consisting of the reaction solution and bacteria. The concentration of bacteria was represented by the viable OD value, which was converted from the tentative OD*, as described earlier.

### 2.3. Rcv Test

**Figure 2:**
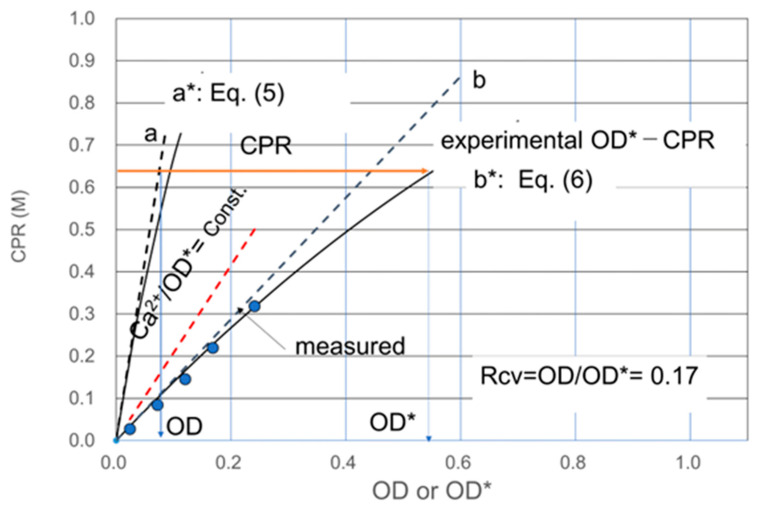
Demonstration of the result of Rcv test, showing the conversion from the OD* axis to the OD axis. a: LSL, maximum tangent of Equation (5), a*: 24 h SC, b: maximum tangent of Equation (6), b*: 24 h OD*-CPR curve.

The CPR measured by using the apparent OD, i.e., OD* is compared with the CPR–OD relationship [16]. The concept and principle are described in previous literature [16], and an example is demonstrated in Figure 2.

Figure 2 demonstrates that the OD value can be converted from Rcv OD*. This relationship is simply represented by the number of alive cells = viability times the total number of alive and dead cells. Therefore, the line of b* in Figure 2 was given by Equation (6). Since the Equation (5) is equal to Equation (6), Rcv (=OD/OD*) value can be obtained by graphical analysis [16] or numerical analysis. Rcv can be determined by the one-point method, which is explained later. It is important to note that in Figure 2, a line and a* line are unique, respectively, but b and b* lines are infinite.

### 2.4. Methods

Test biocement solutions were prepared by a combination of basic OD* and Ca^2+^. Various combinations are needed for different methods of preparation. The preparation method for each case is briefly described in the corresponding section.

The OD* value for the undiluted solution was usually given, or estimated by CPR and Rcv, if known. The volume of bacteria required was calculated by Equation (10). As the mass of bacteria is easier to manage than volume, the volume of bacteria was converted to mass using the density of the concentrated bacteria, 1.07 g/cm^3^. The mass of bacteria was measured with an electronic balance. Note that the concentrated bacteria were diluted by about 1/8000 for preparation of the undiluted solution, in which the weighed bacteria are poured into the mixed solution of other agents or the OD* solution is prepared for mixing with other solution. In this study, the preparation methods are divided into the following three types, i.e., constant Ca^2+^ and various OD*, various Ca^2+^ and constant OD*, and constant Ca^2+^/OD ratio.

## 3. Results and Discussion

### 3.1. Rcv Determination Method of One Point

The experimental results are analyzed using OD, which is converted from OD*. The OD-conversion is described as follows. The OD* values used are tentative, which neglect the effects of unknown factors on the Rcv value such as drainage of bacteria in the supernatant during centrifuging, the effects on Rcv by adding glycerin to concentrated bacteria and unexpected temperature changes. In addition, the ODi and Cf values are not constant, but vary. In this study, both are fixed as 4.0 and 250, respectively, which are approximate averages of the actual data. The OD conversion enables the solving of the problem previously mentioned because OD is expressed by “Rcv OD*”, which can be converted from the one measured CPR for a given OD* at an elapsed time of 24 h.

The standard CPR–OD, Equation (6) is expressed by the following quadratic equation:

17.633 · (Rcv · OD*)^2^ − 8.46 · (Rcv · OD*) − CPR = 0 (11)

The quadratic formula is as follows:

(12) X=8.46−8.46^2−417.633CPR217.633

where X = Rcv · OD* = OD.

Taking the maximum CPR for three evaluations, each Rcv was determined by the following:

Rcv = X/OD*(13)

where X and OD* are known. If Rcv is determined, any OD* can be converted to OD, i.e., Rcv times OD*.

### 3.2. Constant Ca^2+^ Loading

#### 3.2.1. Ca^2+^ of 0.5 M

Reactive solutions of 0.5 M Ca^2+^ were prepared and the various OD* values were adjusted by calculation of V_bac_ in Equation (10). Twelve 10 mL aliquots of different BC solutions were prepared to investigate CPR as a function of OD and time.

**Figure 3:**
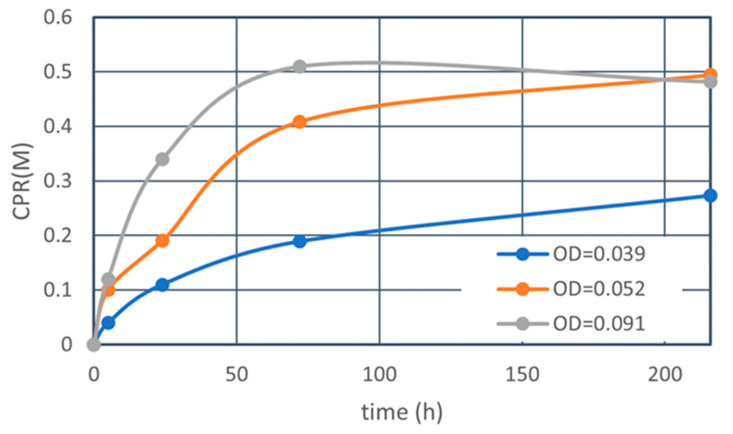
CPR precipitated with elapsed time for different OD values.

The time dependency of CPR was obtained in terms of low OD, as shown in Figure 3. The OD values were converted using the OD* used and the Rcv. The Rcv = 0.31. The CPR was measured by weighing both the dry CaCO_3_ adsorbed in the test tubes and filtered on the paper filters [30]. Thus, the points shown in Figure 3 were determined from different aliquots.

Figure 3 shows that the precipitation rate of CaCO_3_ is higher with a larger OD value. This trend is common [14]. If the maximum CPR is limited by the Ca^2+^ used, it means that the OD value used was sufficient. As the carbonate crystals induced sometimes contain H_2_O, the CPR induced becomes a little greater than the Ca^2+^ used [31,32]. When OD is extremely low, the Ca^2+^/OD is very high. The effects of the high Ca^2+^/OD cannot be neglected.

**Figure 4:**
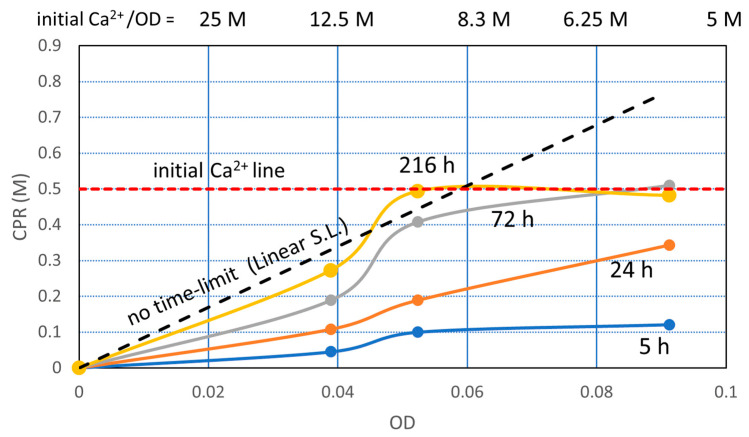
Experimental data and information on fundamental MICP behavior for the initial Ca^2+^/OD load.

The CPR values with OD and time are shown in Figure 4. The trends of OD-CPR curves show that the MICP behavior was governed by the linear standard line (LSL) and the initial Ca^2+^ loading line. As the bacterial cell becomes the nuclei of carbonate precipitation [33,34,35,36], it is assumed that the CPR is governed by the number of viable cells. Zambare et al. [33] summarized that they observed precipitates surrounding bacterial cells through various microscopic methods, and the results supported the hypothesis that bacterial cells can become encapsulated in CaCO_3_ during the MICP process.

As the urease enzyme of bacteria is an embedded type, the products of urease activity, i.e., ammonia and carbon dioxides, are discharged outside of the cell, where calcium ions are abundant [22]. It is possible that the micro-environment of the cell is surrounded by the so-called Stern layer and diffuse double layer, as mentioned earlier. In this study, the number of viable cells is represented by the OD value. Therefore, CPR depending on the number of cells is given by OD. Note that the standard OD-CPR is unique.

Figure 4 shows the OD-CPR curves, after the OD conversion, which is uncommon behavior due to the remarkably high initial Ca^2+^/OD loading rate. The linear standard line (LSL) provides the maximum CPR per OD. In other words, the LSL is the limited capacity of a bacterial cell. The existence of LSL means limit theory of ureolytic bacteria in MICP. Therefore, the Ca^2+^/OD ratio influences the CPR. The software curve fitting would not give the correct curves, particularly between an OD of 0 to 0.04. For example, the CPR at an OD of 0.01 and 0.02 may be lower than Figure 4. It is because the Ca^2+^/OD value increases with the decreasing of the OD value, as indicated at the top of Figure 4.

Thus, the OD-CPR relationships abruptly changed between a Ca^2+^/OD of 8.3 M and 12.5 M because the average capacity of bacteria cells is 8.46 M. The higher the Ca^2+^/OD is, the more damaged the cells are. The inhibition and retardation in the MICP process may be dependent on the degree of damaged cells.

If inhibition or retardation occurs, it occurs at the beginning of the reaction, as the Ca^2+^/OD value is the highest at the beginning. On the other hand, if the reaction occurs at the beginning, it continues until Ca^2+^ is consumed, as the Ca^2+^ concentration necessarily decreases according to the increased CaCO_3_.

The increasing bacteria in the BC solution means that the Ca^2+^/OD ratio decreases, which means less possibility of inhibition and retardation. On the other hand, increasing the Ca^2+^ concentration may not necessarily increase CPR. It increases the risk of inhibition. Note that retardation is not necessarily a risk. The reaction rate is a function of OD, i.e., representative of viable cells. Strictly speaking, the rate is a function of OD, Ca^2+^, pH, Ca^2+^/OD and temperature. The period of 24 h was used to measure CPR, for Rcv and CPR tests, which is defined as 24 h OD-CPR relationship:

CPR = 8.46 · OD − 17.633 · OD^2^

where the first term on the right hand of the equation is for no time limited, and the second term provides the retardation at an elapsed of 24 h.

The time to reach a given CPR for different OD is more important than how much the CPR will be over time.

#### 3.2.2. Ca^2+^ of 1.0 M

Similar test series were performed using an initial Ca ion concentration of 1.0 M. The OD values used were as low as the test series of 0.5 M Ca^2+^.

**Figure 5:**
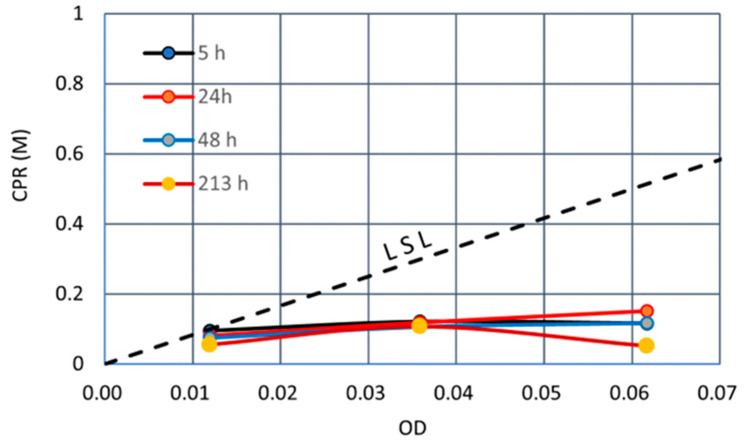
CPRs inhibited under 1.0 M Ca^2+^ load.

The results showed a clear inhibition due to 1.0 M Ca^2+^ load, as shown in Figure 5. Thus, the use of constant Ca^2+^/OD method can decrease Ca^2+^ according to the decreased OD. Therefore, the risk of inhibition can be obtained by this method.

The test results show that the CPRs obtained are 0.1 M for a range of OD from 0.01 to 0.06 and at an elapsed time from 5 h to 213 h. This means that the precipitation stopped at an elapsed time less than 5 h and a concentration of about 0.1 M. The 1.0 M Ca^2+^/OD loading rate is twice that for a 0.5 M loading rate at the same OD values. It may be the reason that inhibition occurred. The details are discussed later.

#### 3.2.3. Low Ca^2+^ Loading (0.3 M)

The 0.5 M and 1.0 M Ca^2+^ loads were examined in the previous sections. Therefore, when a low Ca^2+^ load is examined, it is considered that the whole picture will be revealed. Then, the effects of a 0.3 M Ca^2+^ load was examined using bacteria with Rcv = 0.25. The test solutions were prepared by five levels of OD* concentration. The test solution was prepared and CPRs obtained are presented in Table 1. The CPRs were measured at an elapsed time of 24 h and 72 h.

**Figure 6:**
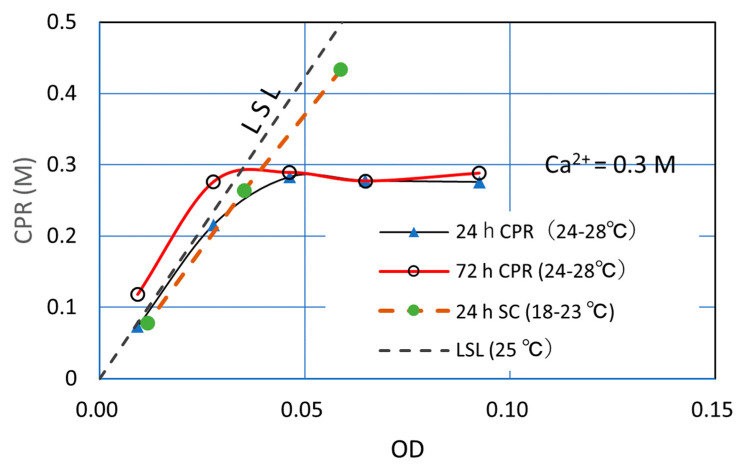
OD–CPR relationship under a 0.3 M Ca^2+^ load.

The results obtained are a little deviated as expected because of the different temperature, as shown in Figure 6. However, the trends are as predicted. First, the CPR was controlled by the Ca^2+^ used. Next, at a low range of OD, the CPR was controlled by bacteria activity, i.e., OD control in contrast to Ca control. The former means that theoretically the products followed stoichiometry. On the other hand, the latter did not follow the chemical equation, but showed a reaction proportional to the viable cell number, which is presented by the linear standard line, LSL.

On the deviation of data, further study remains, which may be the effect of temperature. As shown in Figure 6, the respective tests were conducted at different temperatures. The Rcv test was conducted at temperature 18 to 23 °C. This temperature was a little lower than the temperature of 25 °C at which the standard OD-CPR relationship was evaluated. This may affect the estimated Rcv.

As the temperature affects the precipitation rate CPR, the 24 h CPR for the Rcv test is a little low. For the same reason, the results of 0.3 M CPRs were a little higher than those expected. Particularly, the 72 h CPR test was cured at 28 °C for a long time.

#### 3.2.4. Constant Ca^2+^/OD Ratio

Test solutions of constant Ca^2+^/OD loading rate were used for Rcv and CPR tests [13]. The test solution was prepared by dilution of the originally blended solution with 1.0 M Ca^2+^. The urea concentration was 1.1 M. Therefore, the blend was sufficient to complete the reaction. In fact, the OD conversion shows an excellent fit to Equation (5).

**Figure 7:**
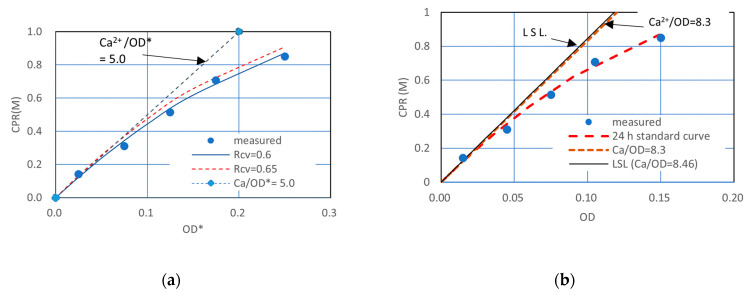
Example of ordinary 24 h CPR curves obtained from Rcv tests (**a**) CPR − OD*, (**b**) CPR-OD.

Similar OD–CPR relationships to Figure 7 were obtained by Lai et al. [14]. The relationships were assumed to be linear. It may be because three measured points were not enough to draw a curve. In comparison, the range of OD used by them is very wide, and the CPRs obtained were low. The different values may result from the different urease activities of the different bacteria [11]. Accordingly, it is considered that the standard OD–CPR relationship varies with bacterial species. The non-linearity by the second terms of Equation (4) is understood as the competitive retardation due to the decreasing Ca^2+^ and increasing CaCO_3_ which was confirmed experimentally, as shown in Figure 7. Note that Figure 7a shows the result of the Rcv test, but Figure 7b shows the OD converted, standard relationship.

In Figure 7, the OD conversion was made by Rcv values obtained from Rcv test, where Rcv was 0.6. The LSL given by 8.46 OD is understood as the competitive-inhibition-free, no time-limited CPR, attributed to the capacity of viable cells. The CaCO_3_ yield to be induced cannot be beyond the LSL. The competitive inhibition is common in enzyme reaction; however, there is no proof of this. The inhibition effects increase with the proceeding of the reaction. Therefore, this case may indicate product inhibition.

Therefore, when the initial Ca^2+^/OD is lower than 8.46 M, all CPR measurements must show the full MICP behavior, which must accord with the standard curve, Equation (5), which is obtained by the OD conversion. Then, all results were obtained at an elapsed time of 24 h, the OD CPR relationship is fitted by the unique 24 h standard curve, as shown in Figure 7b.

**Figure 8:**
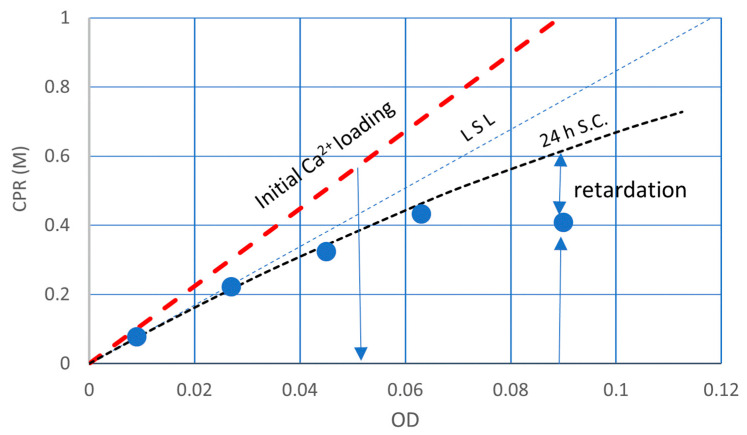
24 h CPR performance at a Ca^2+^/OD value of 11.1, showing 1.0 M Ca^2+^ load under a constant Ca^2+^/OD loading rate.

The different behavior from Figure 7b is shown in Figure 8. The retarded reaction occurred because of the increased Ca^2+^/OD loading rate and will continue until the CPR reaches the LSL because the Ca^2+^ load will decrease due to the decrease in Ca^2+^ by producing CaCO_3_. Accordingly, the highest loading of Ca^2+^ is 1.0 M for the example in Figure 7.

Whether or not the inhibition occurs depends on the Ca^2+^ and OD values, i.e., 1.0 M/OD. It is possible that if the loading rate is less than 8.46 M, no inhibition may occur. In fact, the loading rate Ca^2+^/OD was 11.1. However, the result was not inhibition, but a retarded reaction. By the reaction for 24 h, MICP was 40%. It is expected that the reaction will be complete within 48 h because of the lower loading rate. Note that the retarded point remained as a retarded point after the OD-conversion. Accordingly, when the measurements cannot be fitted by Equation (5) after the OD conversion, it means that the result(s) was retarded or inhibited.

**Figure 9:**
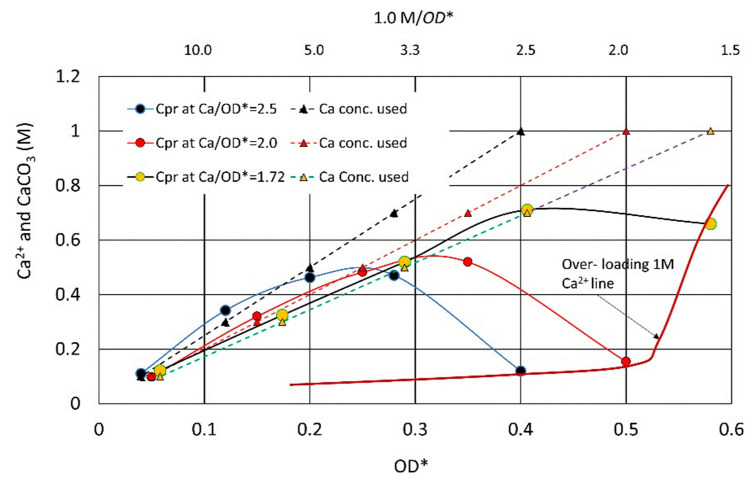
Overload effects due to high load rates [16].

The influence on the MICP behavior due to the increase in the initial Ca^2+^/OD loading rate was evaluated by the OD conversion from the OD*–CPR relationships, which was presented in the previous study [16]. The result showed that the MICP behavior was divided into two patterns, as illustrated in Figure 9. One is the ordinary CPR-producing process along a 24 h standard curve under a lower Ca^2+^ load, and another is the inhibited or retarded MICP behavior, which occurred at a 1.0 M Ca^2+^ load. It was understood that at a 1.0 M Ca^2+^ load, the greater the Ca^2+^/OD or Ca^2+^/OD, the stronger the inhibition and/or retardation are. Thus, the inhibition and retarded behavior are not only influenced by Ca^2+,^ but also OD in terms of Ca^2+^/OD. The details are discussed later. However, constant Ca^2+^/OD load rates mean the increase in Ca^2+^ load for increasing OD values.

In fact, constant low Ca^2+^/OD values have been used for the Rcv tests because CPR can easily be obtained without inhibition of Ca^2+^. In those cases, the CPR–OD* relation was well expressed by Equation (6). The Rcv tests can conveniently be used for determining the optimum blend of the biocement solution.

### 3.3. Discussion

**Figure 10:**
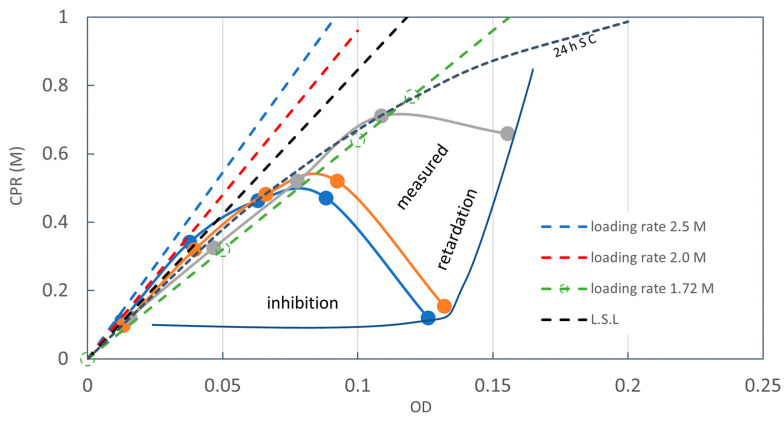
Initial Ca^2+^ conditions used in this study.

It was found that uncommon CPR performances such as inhibition and retardation were influenced by the initial Ca^2+^ concentration, which is the Ca^2+^ loading. In addition, the Ca^2+^/OD loading rate also affects the MICP behavior. The inhibition and retardation for the reaction occur at the beginning because the initial Ca^2+^ load is the highest. For discussion, the OD* axis of Figure 9 was converted to OD axis by the OD conversion. The Rcv values were varied a little for different Ca^2+^/OD*. Therefore, the average Rcv value was used for the conversion. The red results are the converted values of the peak points of CPR for different Ca^2+^/OD* values. It is interesting that these results are on the 24 h SC, as shown in Figure 10.

The initial Ca^2+^ loads used in this study are illustrated in Figure 10. The figure shows that when the Ca^2+^/OD increases, the inhibition zone expands to the left, which accords with Figure 8. The figure also shows that an OD value of 0.118 may be a boundary between inhibition and retardation zones.

**Figure 11:**
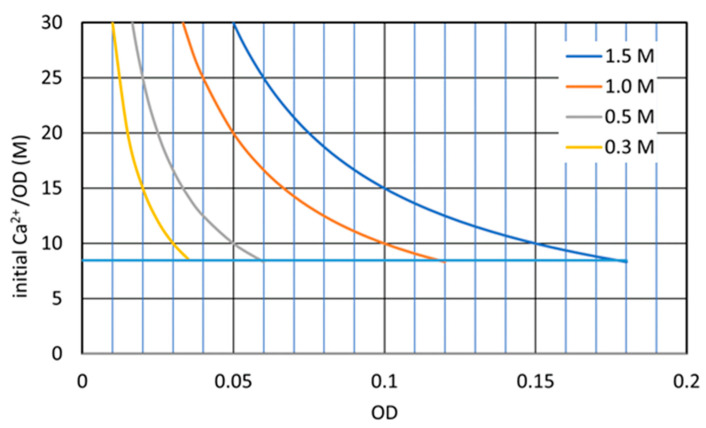
Conditions of inhibited and retarded reaction under 1.0 M Ca^2+^ loading and Ca^2+^/OD loading rates.

From Figure 8 and Figure 9, the overloading effects can be indicated in Figure 11. In a low range of OD, 1.0 M Ca^2+^ seems to be sufficient to cause inhibition. An OD of 0.118, shown by the red broken line, is the limit where the bacteria can induce 1.0 M CPR. The OD value is defined by the linear standard OD–CPR relationship, OD = 1.0 M/8.46 M. Usually, if a half of the full CPR, i.e., 0.5 M is achieved, the reaction can be completed within 48 h. This is because Ca^2+^ concentration decreases with time and the overload decreases.

The different behavior between the 0.5 M and 1.0 M Ca^2+^ loads is due to the effects of intensity of the initial Ca^2+^ load and the initial Ca^2+^/OD loading rate. A load of 1.0 M at low OD showed the clear inhibition, but not for a 0.5 M load. However, the retarded behavior was observed in terms of OD values under 0.5 M Ca^2+^.

**Figure 12:**
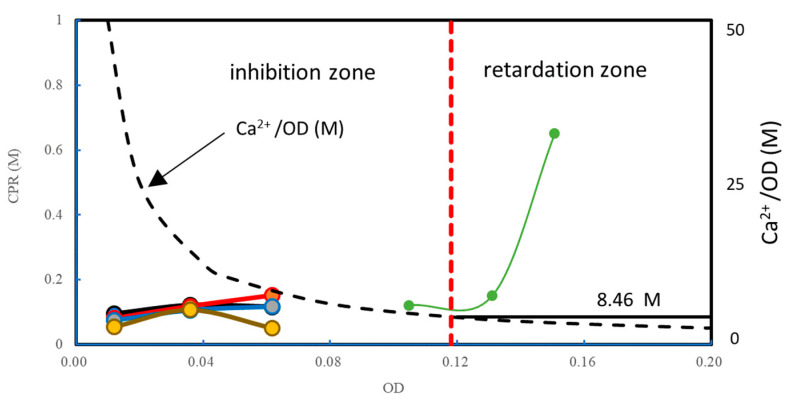
Loading rates for various initial Ca^2+^ loads, as well as boundaries for inhibition and retardation.

Figure 12 shows the changes in theoretical Ca^2+^/OD loading rates under the various Ca^2+^ loadings. In Figure 12, the horizontal line indicates a Ca^2+^/OD = 8.46, which is possibly the tolerance of the biomass. The figure indicates that the higher the initial Ca^2+^ concentration, the OD required to resist the loading effect should be higher. In addition, the lower the OD value, the greater the Ca^2+^/OD loading rate is.

**Figure 13:**
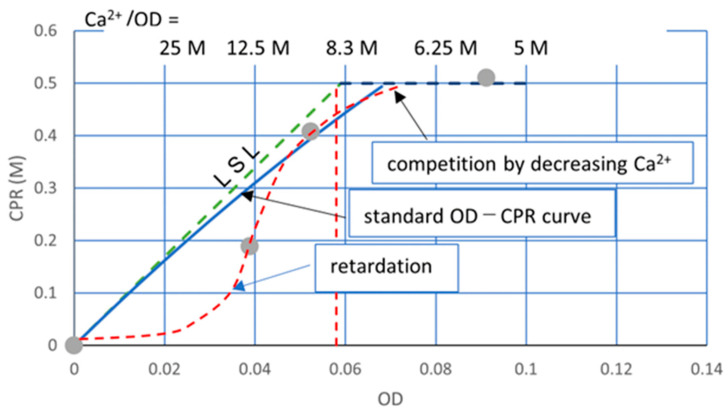
Example of totally retarded reaction at an elapsed time of 72 h used for the OD conversion instead of a 24 h reaction.

Figure 13 indicates a 72 h OD–CPR relationship, which was used for the second estimating Rcv value instead of the 24 h OD–CPR relation, as presented in Figure 4. The result indicates that the reaction was at least a 3-day retardation in comparison to a low loading rate. It is deduced that the retardation is attributed to the Ca^2+^/OD value. In fact, the bacteria at an OD of 0.04 was subjected to a Ca^2+^/OD of 12.5 M, which exceeded 8.46 M, as a critical standard value (Figure 12). Thus, it was found that the MICP behavior was governed by both the initial Ca^2+^ load and the initial Ca^2+^/OD loading rate. Note that the constant Ca^2+^/OD has two meanings, i.e., constant Ca^2+^ and OD values and various Ca^2+^ and OD at the same ratio. The latter involves the change in the Ca^2+^ loading effect.

It is shown in the figure that the inhibition occurred at an OD lower than 0.02, which was given by the dotted line, consisting of the LSL and 0.5 M Ca^2+^ loading lines. It is noted that the Ca^2+^/OD values increase abruptly with a decreasing OD value, as shown in Figure 12. It may be expected that without considering Figure 12, the inhibited behavior was often neglected at an extremely low range of OD values, when, nevertheless, the Ca^2+^/OD was extremely high.

Thus, the disadvantage of a one-point method for determining Rcv appeared in which the CPR is inhibition or retardation. To avoid this, it is useful to take a high OD value for the undiluted test solution. Consequently, the interval of OD values to be used for test tubes can be wide. Note that the minimum OD value is assumed to be always zero. Thus, no test tube for that is required.

For the blending of materials for the BC solution, OD and Ca^2+^ are two primary components. Usually, urea is added by about 10% to the calculated value from the chemical reaction equation. This is a procedure to ensure no calcium is left and to maintain an alkaline condition.

It is considered that the inhibition and retarded behavior in MICP was understood by the established OD-conversion method developed in this study. It is obvious that the standard OD–CPR relationship is different for various species of bacteria because of a wide range in the OD-viability relationship. Therefore, the LSL and 24 h standard curve used in this study cannot be used for other bacteria than NO-A10 [30]. However, the concept and method may be used for any ureolytic bacteria.

The effects of high concentrations of BCS and carbonate on the MICP process was discussed by Lai et al. [14]. They summarized that there was no consensus on the effects of the concentration of the BCS at that time and emphasized that the understanding of retardation effects on MICP is important to determine [14]. Eventually, after investigating the retardation effects on MICP, they concluded that a lower concentration of BCS caused smaller carbonate crystals.

**Figure 14:**
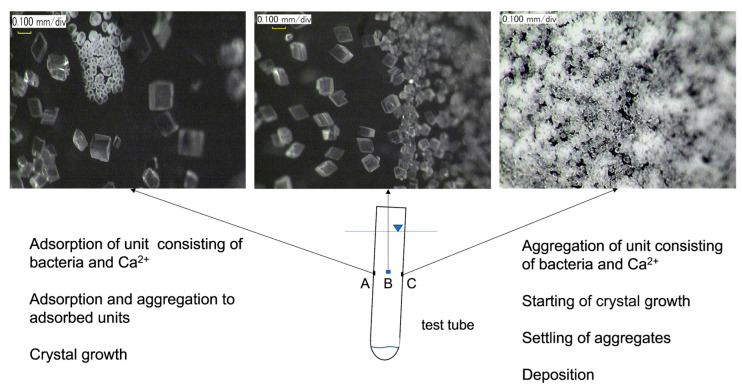
Amorphous cluster and crystalline particles induced in a test tube in the retarded MICP process, at an elapsed time of 7 d under conditions of OD of 0.1 and initial 0.5 M Ca^2+^. Photo by digital microscope, Keyence Co.JP.

The problem of the size of the grains induced is sensitive. A simple observation by a digital microscope showed unpredictable results. The observation was made using a glass test tube. An amount of 10 mL of the test BCS was poured into a test tube and left slightly inclined. The BCS consisted of an OD of 0.2 and 0.5 M Ca^2+^. Seven days later, the photos were taken by means of a digital microscope as illustrated in Figure 14.

The minerals induced on wall A are mostly calcite and partially the cluster of amorphous spherical particles. The size of calcite is about 100 µm and larger calcite particles are also seen. On wall C, the particle’s size is much less than those on walls A and B. It is considered that the morphological difference between the precipitates on walls A and C was attributed to the different MICP behavior if the aggregates were adsorbed slowly on wall A or settled fast on wall C, as demonstrated in the figure. Therefore, the microenvironments for grain growth on walls A and C were different in terms of urease and Ca^2+^. Generally, the greater the number of particles, the lower the grain growth. For example, the photo of wall B showed that the size and number of calcite minerals induced were different along the middle boundary between walls A and C. Thus, the aggregation, adsorption and settling of microbes build the initial structural microenvironments, which affect the following mineral growth.

**Figure 15:**
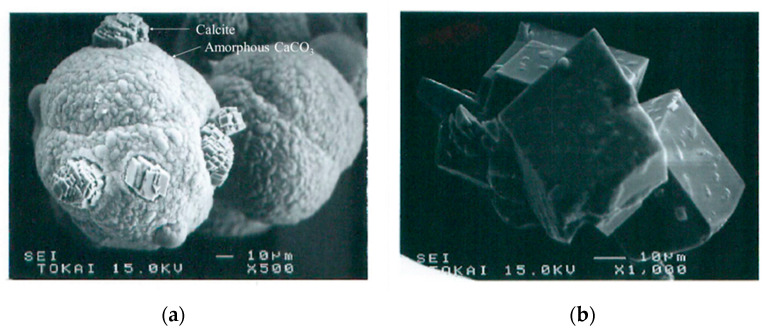
SEM images (**a**) growth of CaCO_3_ crystals from aggregates of bacteria surrounded by amorphous calcites and (**b**) ultimate calcite crystals.

An amount of 100 μm of grain size may require an aggregate consisting of about two thousand bacteria, which hold a large quantity of Ca^2+^. The amorphous calcite may develop around every bacterial cell, and the aggregate grows to approximately 100 μm, as shown in Figure 15. The formation of aggregates has been well studied in colloid science.

The calcite crystals start growing inside of the aggregate, as shown in Figure 15a, while the crystallization will continue forming single or multiple crystals, as shown in Figure 15b. For the formation of smaller crystals, the process is the same.

Lai et al. [14] concluded that the lower concentration of BCS led to the smaller particle size. On the other hand, Mujah et al. [37] concluded that the lower the concentration of BCS, the greater the particle size and that the larger size is more effective in enhancing the strength of soils. These may be due to the complicated MICP behavior, as mentioned.

The low concentration of BCS and low OD value should be combined because of economic and technical points of view. Low OD provides a low reaction rate, which causes a retardation of the reaction. The combination of low OD and high Ca^2+^ causes inhibition and/or retardation. Thus, the combination is given by the Ca^2+^/OD value.

As mentioned, the experimental treatment of bacteria is the hardest problem in the approach of the MICP study. Accordingly, the quality and quantity of bacteria are mostly neglected as factors or parameters. As a result, unknowns are omitted, discussions are impossible, or conclusions are not clear.

In this study, the results shows that the carbonate content increases with an increased concentration of BC solution. On the other hand, it was pointed out that the increase in the Ca^2+^ concentration caused the decreased carbonate content because of the retardation effect [14]. This phenomenon is recognized as the increase in Ca^2+^/OD loading rate in this study.

It was found that the inhibition and retardation were totally dependent on the blending of these two components. To avoid inhibition and retardation, the decrease in Ca^2+^ loading is required, which involves the decrease in OD also from the economic point of view. On the other hand, the decrease in Ca^2+^ must usually increase the volume of the BC solution to maintain the total CPR. This affects the total operation process. Therefore, the ultimate decision must be made from a comprehensive point of view.

In this study, the word “inhibition” is used when the reaction was stopped by the action of inhibitors, while the “retardation” is used when the effect of inhibitors is partial, causing an elapsed time before the reaction is initiated. If the inhibitor is Ca^2+^, it may be possible that thick formation of a diffuse double layer prevents the intake of urea into the cell. This inference supports the association between Ca^2+^ = 8.46 OD and CPR ≦ 8.46 OD, i.e., initial and ultimate conditions for unit bacteria cell. Substituting the concentration of Ca^2+^, the critical OD can be obtained. The critical OD value will give a value of CPR.

The inhibition is the loss of mass to be avoided. However, it is possible that the retardation processes are slow hardening without loss of mass, which influences the morphology of products and affects the hardening due to biocementation.

The correlations between strength and carbonate content using NO-A10 strains have been investigated for many years. Handy, easy-to-use tests such as the pocket, needle and small cone penetration have been conveniently used to investigate the effects of MICP for very loose sandy soils, in which the formation of a specimen cannot be easily performed [38]. In addition, even if the specimen is made, it may fail by tension. However, it seems that many studies have competed on how strong soils can be created, and there were few attempts intended to cure in the order of one hundred kPa unconfined compressive strength.

Furthermore, it was found that the strength of natural marine sediments has been developed by carbonate diagenesis attributed to foraminifers and coccolith [39]. The carbonate diagenesis is not due to biomineralization. However, the results of the ultimate reaction are the same.

The example for the application of this study is given by the blending of BCS for liquefaction measures. Simply, the following relationship is available to determine the OD value:

OD = CPR/8.46 = Ca^2+^/8.46 (14)

where Ca^2+^ (M) is the designed Ca^2+^ (M). Equation (14) has been experimentally confirmed.

**Figure 16:**
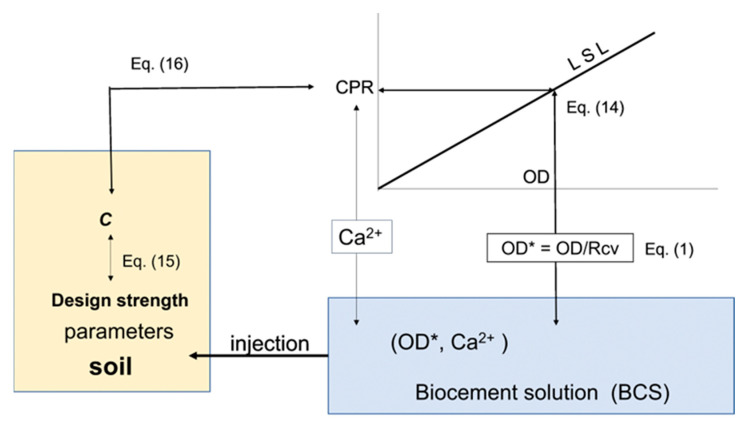
Application flow of LSL, showing association with LSL, BCS and soil.

The design CPR is determined by the design carbonate content, C (%), in which the design strength UCS is as follows:

UCS (kPa) = *a* · C (15)

where *a* is the proportional constant (kPa) depending on the soil type, and C should be lower than five percent. The relation between C and CPR is presented by the following:

C (%) = 10 · n · m/{(1 − n) · rs}(16)

where n is the porosity (decimals) of soil, m is the M concentration, and rs is the dry density (g/cm^3^) of soil particles. Note that Equations (15) and (16) are applied for marine sediments [40]. Saturated flow of BCS is assumed during the injection of BCS. The advantage using this method is that the inhibition is avoided, retardation is allowed, initial Ca^2+^ loading is minimized, and economic design is achieved. The flow chart concerning Equations (14) to (16) is shown in Figure 16.

There is no doubt that the soil strength can be developed by carbonate precipitation. The mass balance between the masses of CaCO_3_ calculated from injected Ca^2+^ and ultimate carbonate content is of importance and should be studied. The inhibition of carbonate precipitation causes the imbalance of masses. As was discussed by Lai et al. [14], there is no consensus if injected Ca^2+^ concentration determines the CPR in soils. There are unknown factors which govern MICP in soils, which may include the existence of LSL, the segregation of bacteria, Ca^2+^ load and inhibition.

## 4. Conclusions

There has been little research on the inhibition of Ca^2+^ on MICP process. This study dealt with the basic interaction properties and behavior for ureolytic bacteria and Ca^2+^ in a urea solution. This manuscript consisted of three main themes. Firstly, the standard OD-CPR relationship was presented, which provides the OD conversion method from aged unknown bacteria, based on previous study. Secondly, OD-CPR relationships under four types of Ca^2+^ loads and constant Ca^2+^/OD loading rates were experimentally examined. Thirdly, the inhibition and retardation in the MICP process were investigated by the OD conversion method and examined to enable CPR prediction.

The main highlights in this study are as follows.

High initial Ca^2+^/OD loading rate and high initial Ca^2+^ load possibly cause the inhibition of MICP. The initial condition to avoid the inhibition is given by Ca^2+^ < 8.46 OD or OD > Ca^2+^/8.46. Thus, the higher the Ca^2+^, the higher OD is required to avoid inhibition and/or retardation. However, in this study, the initial condition Ca^2+^ ≦ 1.0 M should be added because no experimental data for Ca^2+^ is higher than 1.0 M. The optimum blending condition is given by Ca^2+^/OD = 8.46.

Considering OD is a function of the viable number of cells, the condition to avoid inhibition is given by the function of viable cell number. In other words, the condition given is due to the ability of the cell or the limit or capacity of urease activity.

The retardation should not be avoided because the slow reaction usually contributes to a higher strength of soils unless a shorter time frame is required. If the CPR at 24 h is about half of the full precipitation, the ultimate precipitation usually reaches full precipitation. The retardation condition is included in avoiding inhibition.

The OD conversion is enabled to compare the experimental data obtained using aged bacteria. This can give knowledge about MICP. In addition, there may be the possibility that by coordinate transformation, the OD*–CPR relationship obtained by using any species of bacteria can be converted to the standard OD–CPR relationship proposed in this study. Future work will examine the relationship between the unconfined compressive strength of biocement-treated soils and carbonate content, which can be estimated from CPR, and also the dry density of the soil as an application to civil engineering is the ultimate objective.

## Figures and Tables

**Table 1:** Test solution and CPRs obtained.

Ca^2+^	OD*	OD	24 h CPR	72 h CPR
(M)			(M)	(M)
0.3	0.370	0.093	0.276	0.288
0.3	0.259	0.065	0.278	0.277
0.3	0.185	0.046	0.283	0.289
0.3	0.111	0.028	0.216	0.276
0.3	0.037	0.009	0.073	0.118

## Data Availability

Data available on request.
